# The relationship between problematic gambling severity and engagement with gambling products: Longitudinal analysis of the Emerging Adults Gambling Survey

**DOI:** 10.1111/add.16125

**Published:** 2023-01-31

**Authors:** Heather Wardle, Sarah Tipping

**Affiliations:** 1 School of Social and Political Sciences, University of Glasgow, Glasgow, UK; 2 Faculty of Public Health and Policy, London School of Hygiene and Tropical Medicine, London, UK; 3 Sarah Tipping Consultant, Lingfield, UK

**Keywords:** Covid-19, emerging/young adults, gambling, harms, longitudinal, products

## Abstract

**Aims:**

To measure the association between problem gambling severity and 19 different gambling activities among emerging adults (aged 16–26).

**Design:**

An online non-probability longitudinal survey collecting data in two waves: wave 1, July/August 2019; wave 2, July/October 2020.

**Setting:**

Great Britain

**Participants:**

A total of 2080 young adults participating in both waves.

**Measurements:**

Problem gambling scores were collected using the Problem Gambling Severity Index (PGSI). Binary variables recorded past year participation in 19 different gambling forms, ranging from lotteries to online casino and gambling-like practices within digital games (e.g. loot box purchase, skin betting). Controls included socio-demographic/economic characteristics, the Eysenck Impulsivity Scale and the number of gambling activities undertaken.

**Findings:**

Zero inflated negative binomial model lacked evidence of an effect between past year participation in any individual activities and subsequent PGSI scores. However, negative binomial random effects models for current gamblers (n = 497) showed that skin betting (incidence-rate ratio [IRR] = 2.32; 95% CI = 1.69–3.19), fixed odd betting terminals (IRR = 2.21, 95% CI = 1.61–3.05), slot/fruit machines (IRR = 1.43, 95% CI = 1.07–1.91), online betting on horse/dog races (IRR = 1.53, 95% CI = 1.17–2.00) and online betting on non-sports events (IRR = 1.44, 95% CI = 1.11–1.89) were associated with increased PGSI scores. Online casino gambling had a significant interaction by wave; the impact of online casino betting in wave 2 on PGSI scores increased by a factor of 1.61.

**Conclusions:**

Past year participation of young adults (aged 16–26) in certain forms of gambling does not appear to be associated with future Problem Gambling Severity Index scores. Among young adults who are current gamblers, past year participation in certain land-based (e.g. electronic gaming machines) and online forms (e.g. skin betting) of gambling appears to be strongly associated with elevated Problem Gambling Severity Index scores.

## Introduction

Understanding the relationship between certain forms of gambling and gambling harms is a critical policy consideration. In Britain, legal forms of gambling range from lotteries to electronic gaming machines (EGMs) and online casino products. As each form of gambling is associated with a different set of structural characteristics, and are provided in different contexts, debate has focused on the extent to which each type may be more (or less) harmful and their consequent association with problematic gambling [1–4]. Engagement in continuous forms of gambling activities, like EGMs or online equivalents, has been consistently associated with gambling problems [3].

A range of potentially interlinked explanations have been suggested for these patterns. These include selection effects, whereby those with greater vulnerability gravitate to certain forms of gambling; exposure effects, whereby those exposed to certain forms of gambling are at greater risk because of the structural characteristics of the gambling format and broader commercial effects, whereby the commercial actions of the industry, especially pertaining to access, availability, marketing and promotion, increase risk propensity [3, 5, 6]. Conversely, some have argued that the range and breadth of gambling involvement, rather than engagement in specific activities, are more useful predictors of harms (termed the involvement hypothesis) [4].

Yet few studies have focused on the relationship between engagement in certain forms of gambling and problematic gambling among the ‘emerging adult’ age cohort specifically, although associations have been noted between online gambling and problematic gambling for young men and scratchcard purchase and problematic gambling [7–9]. Given the changing landscape of gambling provision in Britain, and continuing debate about the role of gambling-adjacent activities (e.g. loot boxes) in the production of gambling harms [10–12], this warrants attention to map key associations and empirically test some of the suggested mechanisms underpinning observed associations (such as selection effects etc).

Emerging adults (those approximately 18–24 years old) have been identified as a group at heightened risk of experiencing problem gambling. Forrest and McHale [13] showed that rates of problem gambling increased significantly between the ages of 17 and 21, leading them to suggest that extra measures could be warranted to protect emerging adults from harms during this period of increased vulnerability. This was a key question posed by the British government in their review of the 2005 Gambling Act [14]. Furthermore, according to Arnett [15], who coined the term ‘emerging adult’, this age group are demographically distinct with a greater propensity for risk-taking behaviour, including impulsivity, and engaging in sensation-seeking experimentation before settling into adult roles and responsibilities [15]. These are known risk factors for the experience of problem gambling.

This study explores how gambling activities, but also newly emerging gambling-adjacent activities within digital games, are associated with problem gambling severity. Loot boxes are one example of ‘gambling-adjacent’ activities, described as psychologically akin to gambling [16]. They are items that may be bought for real-world money containing randomized contents whose value is uncertain at the point of purchase [17]. The betting of skins is another example. Skins are decorative items bought or won within digital games that can be traded or bet through a series of connected marketplaces [18]. Because skins can be used as collateral for betting on various websites and because loot boxes mimic variable reward mechanisms has led to wide-scale debate about whether these represent new forms of gambling [18]. Few studies have measured both gambling-adjacent and for-money gambling activities concurrently, although researchers have increasingly argued they should be considered in parallel [19].

Using a longitudinal survey of emerging adults (aged 16–26) this paper conducts exploratory analysis to:

- explore the relationship between problem gambling scores and engagement in different forms of gambling (including gambling-adjacent activities: loot box purchase; skin betting), while controlling for demographic/socioeconomic status and gambling involvement, and,
- examine if and how these associations changed over time.

## Methods

### Design

The Emerging Adults Gambling Survey is a longitudinal study of young people aged 16 to 26 living in Britain. The study’s primary aim was to examine individual gambling trajectories over time; sample size calculations were based on being sufficient to estimate change in gambling behaviours between waves. Assuming a between wave correlation of 0.5, the study was designed to be able to detect changes in problem gambling behaviours of ±0.3 percentage points (at 80% power). The survey protocol was pre-registered [20]. Data analysed here include participants from wave 1 (*n* = 3549) and wave 2 (*n* = 2080). Participants were drawn from YouGov’s online panel of over 1 million people living in Britain [21, 22]. Participants were eligible if they were aged 16 to 24 (at wave 1), living in Britain and had not participated in another YouGov study on gambling in the past year at baseline. E-mail invitations were sent by YouGov to selected panellists inviting participation in a survey, without advertising its content, and to click through to the bespoke study. The first page of the bespoke survey described the study’s aims and objectives, including that this was a longitudinal survey and that we would be wishing to recontact them 1 year later and obtained consent. In wave 1, 93% of people who accessed this page went on to complete the survey. Participants received YouGov points (equivalent to 50p in value) for taking part. All participants from wave 1 were recontacted by YouGov a year later and asked to take part. Wave 1 data were collected between 25 June 2019 and 16 August 2019. In wave 2, 2080 participants of 3549 took part, representing a retention rate of 58.6%. Data were collected between 13 July 2020 and 8 October 2020. Wave 2 data was collected during the coronavirus disease 2019 (COVID-19) pandemic, where lockdown conditions in Great Britain saw the closure of most land-based gambling venues between 23 March 2020 and July 2020 and the cancellation of live sports events between March 2020 and June 2020.

The questionnaire covered gambling, gaming, social media and health-related behaviours; it was developed by the lead author and reviewed by an expert panel. Before wave 1, an online pilot collected data from 62 participants in May 2019. These responses were reviewed by the lead author and YouGov and changes agreed. In both waves, the first 250 responses from mainstage data collection were reviewed for consistency, routing accuracy and to establish timing thresholds for seriousness checks. Participants who completed the survey in less than one SD of the mean completion time were removed (<2 min and 30 s for non-gamblers; <4 min for gamblers): 39 participants (wave 1) and 14 participants (wave 2) were excluded for this reason. Missing data because of item non-response were minimal and excluded from analyses (except where explicitly stated, see Supporting information Appendix S1). (See Supporting Information Appendix S3 for the Strobe Checklist for Cohort Studies).

## Measures

### Outcomes

Problem gambling was measured using the Problem Gambling Severity Index (PGSI), a validated tool for the identification of gambling problems [23] (wave 1 alpha: 0.79; wave 2 alpha 0.79). This was asked of anyone who had gambled in the past year and produces a score ranging from 0 to 27.

### Exposures

In both waves, participants were asked to report participation in the last 12 months on a range of 17 gambling activities legally available to those ages 18 and over in Britain.* These data were coded into a series of binary variables showing if a participant had engaged in each in the past year (1) or not (0). Participants were asked how often in the last 12 months they had paid money to open loot boxes when playing video games or how often they had bet in-game video game items (e.g. skins) on either external websites or privately. Binary variables showing participation in the past year (1) or not (0) were generated for these activities.

### Controls

Impulsivity was measured using a shortened form of the Eysenck Impulsivity Scale validated for use among adolescents [24–26]. Responses to seven impulsivity statements were recorded on a five-point scale with response options ranging from very true (1) to not at all true (5). Impulsivity scores were computed as the average of the seven questions. Impulsivity was collected at wave 1 only and scores used as fixed effects in the model (wave 1 mean [SD] = 2 × 28 [0 × 87]).

Ethnicity was reported in wave 1 using the United Kingdom’s (UK’s) Office for National Statistics harmonised ethnic group question. This lists 18 possible ethnic group codes. Because of low base sizes, responses were grouped into White vs non-White. Missing values were coded to the modal category: White. Age was captured in single age years. Region of residence and local area level deprivation at wave 2 was measured using English, Scottish and Welsh Indices of Multiple Deprivation (IMD) scores matched at the ‘Output Area’ and quintiled for analysis. Participants reported both their own level of academic attainment (at wave 2), which was grouped into: had degree; did not have degree, and their parents’ level of academic attainment. Responses were grouped by whether at least one parent had a degree or higher or whether both parent’s qualifications were lower than degree level. Marital status at wave 2 was asked and grouped into: lives with a partner; has partner, but does not live with them; single. Finally, personal gross income at wave 2 was obtained and grouped by: <£5000; £5000–<£20 000; £20 000 or more. Missing data for income was high and coded as a dummy category.

## Analyses

Frequencies described the characteristics of the sample and participants gambling behaviours (Tables 1 and 2). The relationship between gambling activity and PGSI score was first explored using zero-inflated negative binomial models (Table 3). The model uses wave 2 PGSI score as the dependent variable and a set of binary indicators showing participation in each gambling activity (including skin betting and loot box purchase) in the past 12-months at wave 1 as predictors. The model indicates whether participation in a specific activity at wave 1 is associated with the PGSI score at wave 2, when controlling for gambling involvement (measured by the number of activities undertaken); other demographic/socioeconomic controls and PGSI score at wave 1.

The relationship between participation in each gambling activity and PGSI score was further explored for those who gambled at both waves using negative binomial models. This approach looks at the associations between current gambling activities and current PGSI scores. It also describes whether the nature of this relationship, in terms of direction and magnitude, changed over time. Unlike the zero-inflated models, the PGSI score used as the dependent variable in this model is for gamblers only and does not contain excess zeros, hence the first step of the modelling—the logit model used to estimate whether or not a zero PGSI score was because of an absence of gambling—is not required. The remaining count model is a negative binomial model. This less complex approach allows random effects to be included in the model to account for the panel structure of the data, incorporating a longitudinal element into the analysis.

Data were transformed into ‘long’ format for this model (meaning each row of data is a subject at a particular time point; individuals who responded to both waves have two data entries, those who responded to a single wave only have one). The model uses PGSI score (the long data format means this is across both waves) as the dependent variable and participation in each gambling activity (including skin betting and loot box purchase) as predictors. Survey wave was also included as a predictor. Interaction terms between the different activities and survey wave were used to identify whether the relationship between the activity and PGSI scores altered between waves. These models include those who gambled at both wave 1 and wave 2 (*n* = 497 individuals, with 994 data entries) (Table 1, column b).

A negative binomial random effects model was preferred over a Poisson regression because the PGSI scores for gamblers were over-dispersed, with a mean of 1.9 (for those who gambled in both waves) and variance of 20.9, meaning Poisson regression would underestimate the standard errors and inflate the associated test statistics [27, 28].

Random effects models account for the longitudinal data design with repeated measures taken from the same individual over time. The model assumes independence exists between the different individuals in the sample, but not between time points for the same individual and that the over-dispersion in the dependent variable varies between individual, but is consistent within individuals over time. These assumptions were tested using a likelihood-ratio test. A significant test result (*P* < 0.05) confirmed these assumptions.

Two regression models were run. The first (Table 4a and 4b) included information on all 19 gambling activities (Table 2). Interaction terms between each activity and survey wave were included in the regression (Table 4a). The second model (Table 5a and 5b) was more parsimonious containing only activities and interactions with *P* values <0.05 from model 1. Activities with *P* values >0.05 from model 1 were removed from model 2 following a stepwise procedure, whereby the least significant terms were dropped (Table 5a). At the removal of each term the model containing the remaining terms was assessed before further terms were dropped. If an interaction term was significant then the main term was retained.

The covariates to be included in the parsimonious model were reviewed using Akaike’s information criteria (AIC), rather than *P* values, as the basis for inclusion. This metric is based on the model’s log likelihood and penalises free parameters in the model. It was used to compare nested models and assess whether additional covariates constitute an improvement to the model. Starting with a model containing the control variables only, activities were in turn entered into the model and the values for AIC compared. The covariate that improved AIC the most was then included in the model and the process repeated. This approach resulted in the same model as the parsimonious model.

Bayesian information criterion (BIC) was used in tandem to review the models. Although BIC would suggest a more parsimonious model (excluding slots, online casino, other online betting and both interaction terms), we felt the evidence from AIC (delta AIC ranged between 2.6 and 6.1 on these additional covariates) was sufficient for us to include the terms given BIC is known to underfit and the additional covariates were both interpretable and made theoretical sense.

For both models, a standardised set of controls were included (see measures). The gambling activities were tested for collinearity by calculating variance inflation factors (VIF); all VIF values were <2 [29].

For each model coefficient a Z-test is produced as the default test of significance. This tests the extent to which the outcome variable (PGSI score) varies by the dependent variables (the gambling activities) and is the test on which the *P* values for each coefficient are based. All analysis was run using Stata 17.

The data are affected by survey non-response and panel data attrition (attrition between wave 1 and wave 2 was 41.4%). Weights were generated to address non-response bias (see Supporting information Appendix S2). Analyses in Tables 1 and 2 are weighted; true (unweighted) bases are also presented. Weights were not applied to the regression analysis. Random effects models are not designed to be run on weighted data; the weights cause the estimates of the random effects variances to be biased. Instead, the regression models control for a range of socioeconomic and demographic variables, ensuring the reported relationships between gambling activities and PGSI score are not affected by biases in these factors resulting from attrition.

To test the sensitivity of the models, data were split at random into two halves and the modelling repeated. Despite the low prevalence of some gambling activities and smaller sample sizes, the IRRs from both were similar in direction and magnitude to the full sample (see Supporting information Tables S1 and S2).

## Results

### Characteristics of the sample

Tables 1 and 2 show the socio-demographic and gambling characteristics of the sample for each wave individually (column a) and for those who gambled in both waves (column b). Overall, 43.4% (95% CI = 41.1–45.8) of participants had gambled on any activity in the past 12 months in wave 1 and 34.0% (95% CI = 31.8–36.3) had done so in wave 2. The reduction in past year gambling activity between wave 1 and wave 2 is likely related to the impact of COVID-19 on gambling supply during that period.

The most common activities undertaken by those gambling in both waves (n = 497) were: lotteries (49.8%, 95% CI = 46.4–53.2); scratchcards (47.7%, 95% CI = 44.3–51.1); online sports betting (32.5%, 95% CI = 29.3–35.8); online betting on horses/dog races (18.0%, 95% CI = 15.5–20.8) and loot boxes (17.6%, 95% CI = 15.1–20.5). Mean PGSI scores were 1.9 (SD = 4.57).

### Multi-variate analyses

Table 3 show the IRRs between past year engagement in each gambling activity at wave 1 and wave 2 PGSI scores, controlling for gambling involvement, socioeconomic status, demographics and impulsivity. Results lacked evidence of an effect between past year participation in any individual activities and subsequent PGSI scores, with the exception of private betting where engaging in this at wave 1 decreased PGSI scores at wave 2 (IRR = 0.56, 95% CI = 0.36–0.86). This pattern was true regardless of whether number of gambling activities was included as a control or not (results available at https://osf.io/6sem8).

Looking at current gamblers only, Table 4a shows that past year gambling on fixed odd betting terminals (FOBTs), skin betting, online betting on horses/dog, online casino/slot gambling, playing poker at a pub/club; purchasing loot boxes or playing fruit/slot machines were each significantly related to having a higher PGSI score across both waves.

Two interactions terms for individual activities in Table 4a were significant (*P* < 0.05). These were lotteries and playing poker in a pub/club, suggesting that the relationship between these specific activities and PGSI scores changed between the two waves. With respects to lotteries, the *P* value for the main effect was 0.705, (IRR = 1.07, 95% CI = 0.76–1.49), yet the interaction term was significant (*P* = 0.001). The IRR for the interaction term was 0.51 (95% CI = 0.34–0.76) suggesting that impact of playing the lotteries on PGSI scores decreased at wave 2 by nearly half. By contrast, the main effect for poker was that playing poker at wave 1 increased PGSI scores by a factor of 1.99. However, interaction term showed an IRR of 0.48 (95% CI = 0.24–0.97). The impact of playing poker on PGSI scores, therefore, changes by a factor of 0.48 at wave 2. In short, there was a positive relationship between playing poker and PGSI at wave 1 that had weakened by wave 2. For all other activities, the interaction terms suggested the associations remained similar between waves.

Table 5a shows results for the more parsimonious model, where activities were only retained if significant (loot boxes purchasing and poker were removed from the model under this process). As Table 5a shows, skin betting, playing FOBTs, playing slot/fruit machines, online betting on horse and dog races and online betting on things other than horses/sports were associated with increased PGSI scores. Skin betting had the strongest relationship to PGSI, increasing scores by a factor of 2.32 (95% CI = 1.69–3.19). FOBTs (IRR = 2.21, 95% CI = 1.61–3.05), online horse betting (IRR = 1.53, 95% CI = 1.17–2.00), other online betting (IRR = 1.44, 95% CI = 1.11–1.89) and playing fruit/slots machines (IRR = 1.43, 95% CI = 1.07–1.91) were all associated with increased PGSI scores.

There were two significant interaction terms: lotteries and online casino/slot games. The pattern for lotteries was similar to findings observed in Table 4a, whereby playing lotteries at wave 2 decreased PGSI scores by a factor of 0.69 is (*P* value = 0.032, IRR = 0.69, 95% CI = 0.49–0.97). Looking at online casino/slot games, the *P* value for the main term was *P* = 0.145 (IRR = 1.26, 95% CI = 0.92–1.73) whereas the *P* value for the interaction term was *P* = 0.009 (IRR = 1.61, 95% CI = 1.12–2.30). This suggests that impact of playing in online casinos in wave 2 on PGSI scores increased by a factor of 1.61, indicating that betting in online casinos has a larger impact on PGSI scores in wave 2 than wave 1.

## Discussion

Analysis looked at the associations between gambling activities and PGSI scores in two ways. First, among all participants, we looked at the extent to which past year engagement in certain activities was predictive of future PGSI scores. This showed no clear discernible pattern for any individual activity. Past year participation may be too blunt a measure to determine this or other measures, such as frequency or depth of gambling engagement may perform better when it comes to the identification of future PGSI severity. Equally, disruption to gambling supply during wave 2 because of COVID-related lockdown conditions and the cancellation of live sports events may have generated changes in subsequent gambling behaviours and problem gambling severity not ordinarily apparent. A study of British sports bettors showed that faced with restrictions on sports betting opportunities, approximately a third stopped gambling [30]. Gambling Commission data shows that fewer people gambled during the initial stages of the pandemic and relatedly rates of problematic gambling fell [31]. Therefore, disruptions to gambling supply experienced between wave 1 and wave 2 may have resulted in more young people stopping gambling than usual, which in turn may impact on the associations observed between wave 1 activity participation and PGSI score at wave 2.

However, focusing on analysis among current gamblers revealed interesting patterns. Among emerging adults in Britain, gambling on online horse/dog races and other events, and playing EGMs (both slots and those formerly known as FOBTs) were associated with elevated problem gambling scores.

The results for EGMs are not surprising. They have routinely been associated with elevated rates of problem gambling among adult populations and this data replicates this finding among emerging adult gamblers [32, 33]. EGMs are a continuous form of gambling, highlighted as one of the strongest risk factors for problem gambling among all adults [3, 32]. The findings for FOBTs are notable, given data were collected after the British government reduced the maximum stake sizes on these machines to £2. The continuing association between FOBTs and elevated problem gambling scores among gamblers of this age suggests that this relationship has not been fully mediated by this policy change. Although, in Britain, much policy attention has been given to online forms of gambling, especially with the British Government’s pending review of the Gambling Act 2005 [14], these results highlight the importance of continuing to address harms associated with land-based forms of gambling, especially EGMs.

Notably, analyses for current gamblers took into account broader gambling involvement and a range of socioeconomic and demographic vulnerabilities as well as impulsivity. This suggests that factors other than gambling involvement or vulnerable groups being attracted to these forms may explain the association between these gambling forms and problem gambling severity. This might include the structural characteristics of these gambling formats and/or commercial or regulatory practices governing their provision and promotion.

With respect to changing behaviours over time, the significant interaction term for online casino/slot style games is notable. Although associations between online casino/slot engagement and PGSI scores were statistically inconclusive at wave 1, the impact of playing in online casinos in wave 2 on PGSI scores increased by a factor of 1.61, indicating that betting on online casinos has a larger impact on PGSI scores in wave 2 than wave 1. Wave 2 data was collected in July–October 2020, during the COVID-19 pandemic. During this time, online gambling firms, particular online casino/slot games reported growth in the number of active players and in revenues [34]. Concern was raised about the potential for some people to engage more problematically with these products during this time [35]. The observed interaction in this study may reflect these broader processes.

Finally, the largest association was observed for skin betting. To date, much attention has been given to the relationship between loot boxes and problem gambling scores, with less focus on other gambling-like mechanics within the digital game ecosystem [11, 12, 16, 17]. Skin betting involves using items from digital games as collateral to wager. Our results show that emerging adult gamblers who engaged in these practices increased their PGSI scores by a factor of 2.32. Few studies have examined loot boxes and skin betting simultaneously. In our study, loot box gambling was significant in the first, but not the second, more parsimonious model. This raises questions about whether associations observed between loot box gambling and problem gambling scores are driven by other factors, for example, concurrent engagement in skin betting. This needs further investigation.

In summary, our results tentatively suggest that focus on past year participation in certain forms of gambling during the disruptive period of the COVID-19 pandemic are not a good predictor of future PGSI scores. However, in the case of current gamblers, past year participation in certain forms of gambling, like EGMs or skin betting, are positively associated with concurrent problem gambling severity, and in the case of online casino/slots this association may be strengthening over time.

This study has a number of limitations. First, the YouGov panel is a non-probability sample with attendant issues of generalisability. Nevertheless, compared with other sample frames, it has good sample coverage, including young people both in and out with full time education (unlike sample drawn from Higher Education Institutes or the Postcode Address File, which excludes those living in halls of residences). Studies have shown that although online non-probability methods should not be used for prevalence estimates, they can perform better (although still not without some issues) when focusing on the relationship between variables, which this study does [36]. Second, attrition between waves was high, although commensurate with other longitudinal studies of young people [13]. Relatedly, because of attrition, the sample size was smaller than hoped. Engagement in some of the forms of gambling reported here are relatively rare. Therefore, non-association should not be taken to mean that these things are not related, but rather that the study was underpowered to examine these. Finally, this analysis focuses on whether people had participated in gambling in the past year or not. It would be useful to explore how changes in gambling frequency for each activity also relate to problem gambling scores. Because of changes to the wave 2 questionnaire necessitated by the COVID-19 pandemic, it was not possible to include this here. Future studies should assess this.

## Conclusion

Among current gamblers, both land-based and online forms of gambling were strongly associated with elevated PGSI scores among young adults. Skin betting, rather than the purchase of loot boxes, emerged as one of the strongest predictors of elevated PGSI scores. Although policy attention should focus on online gambling and gambling-adjacent forms, EGMs in land-based venues should continue to command attention.

## Supplementary Material

S1

S2

S3

## Figures and Tables

**Table 1:** Participant profile.

	*a. All participants*						*b. Those who gambled in both waves*					
	*Wave 1*		*Wave 2*		*Combined*		*Wave 1*		*Wave 2*		*Combined*	
	*n*	*%*	*n*	*%*	*n*	*%*	*n*	*%*	*n*	*%*	*n*	*%*
Gender^a^												
Female	1268	46.4	1268	46.4	2536	46.4	283	41.2	283	41.2	566	41.2
Male	812	53.6	812	53.6	1624	53.6	214	58.8	214	58.8	428	58.8
Age												
Mean age:	2080	20.5	2080	21.5	4160	21	497	21.2	497	22.2	994	21.7
SD		2.4		2.4		2.5		2.3		2.3		2.4
Ethnic group^a^												
White	1822	86.8	1822	86.8	3644	86.8	449	89.8	449	89.8	898	89.8
Non-White	258	13.2	258	13.2	516	13.2	48	10.2	48	10.2	96	10.2
Tenure												
Own with mortgage or part own scheme	299	16.7	277	15.7	576	16.2	76	16.5	73	16	149	16.3
Rent from local authority/housing authority	679	31.3	691	31.1	1370	31.2	173	33.1	183	34.7	356	33.9
Live with parents/family/friends-pays rent	324	16.3	374	18.4	698	17.4	104	23.5	118	24.7	222	24.1
Live with parents/family/friends-does not pay rent	778	35.7	738	34.8	1516	35.3	144	27	123	24.6	267	25.8
Marital status												
Live with partner	305	12.2	354	14.4	659	13.3	111	18.1	121	19.7	232	18.9
Do not live with partner	446	20.4	410	19.6	856	20	112	22.3	104	21.8	216	22
Single-no partner	1329	67.4	1316	66.1	2645	66.7	274	59.6	272	58.5	546	59.1
Employment/education status												
In education/employment or training	1802	87.7	1664	80.9	3466	84.3	451	92	423	86.5	874	89.2
Not in education/employment or training	278	12.3	416	19.1	694	15.7	46	8	74	13.5	120	10.8
Personal income												
<£5 k	605	31	522	27.5	1127	29.3	106	22.2	83	17.6	189	19.9
£5 k-£19 999	442	20.1	439	20	881	20.1	139	27	134	25.9	273	26.5
£20 k+	310	14.9	418	19.6	728	17.3	120	24.4	155	30.5	275	27.4
Unknown	723	33.9	701	32.9	1424	33.4	132	26.4	125	26.1	257	26.2
Educational attainment												
Does not have a degree or higher	1398	69.4	1265	63.4	2663	66.4	283	59.3	263	55.5	546	57.4
Has a degree or higher	682	30.7	815	36.6	1497	33.6	214	40.7	234	44.5	448	42.6
Parental education^a^												
Degree or higher	1196	57.5	1196	57.5	2392	57.5	260	52.6	260	52.6	520	52.6
Secondary education	736	35.4	736	35.4	1472	35.4	209	41.8	209	41.8	418	41.8
Primary or lower	24	1.2	24	1.2	48	1.2	6	1.2	6	1.2	12	1.2
Unknown	124	5.9	124	5.9	248	5.9	22	4.5	22	4.5	44	4.5
Region												
North East	70	3.7	65	3.6	135	3.6	19	4.0	17	3.9	36	4.0
North West	211	10.3	214	10.3	425	10.3	59	12.1	59	11.6	118	11.9
Yorkshire and the Humber	190	8.7	188	8.8	378	8.7	51	8.5	54	9.3	105	8.9
East Midlands	145	7.7	140	7.4	285	7.6	28	5.5	27	5.2	55	5.3
West Midlands	170	8.7	172	8.8	342	8.7	42	9.6	43	9.7	85	9.7
East of England	174	9.7	175	9.7	349	9.7	41	10.1	43	10.4	84	10.2
London	305	13.4	317	13.8	622	13.6	62	11.6	65	11.9	127	11.7
South East	296	13.0	293	13.1	589	13.0	72	13.0	71	13.2	143	13.1
South West	204	8.1	208	8.3	412	8.2	35	6.2	34	6.2	69	6.2
Wales	96	5.7	89	5.3	185	5.5	32	8.6	29	7.7	61	8.1
Scotland	184	8.2	184	8.1	368	8.2	47	8.3	46	8.0	93	8.2
Northern Ireland	35	3.0	35	3.0	70	3.0	9	2.7	9	2.8	18	2.8
Area deprivation quintile												
Unknown	169	14.5	213	16.3	382	15.4	22	7.6	28	8.9	50	8.3
1st least deprived	383	17.1	381	17.1	764	17.1	94	17.7	90	17.0	184	17.4
2nd	397	16.3	366	15.4	763	15.8	97	17.5	97	17.6	194	17.5
3rd	388	16.7	373	16.1	761	16.4	86	16.2	85	15.9	171	16.1
4th	357	17.4	374	17.6	731	17.5	85	17.1	87	17.1	172	17.1
5th most deprived	386	18.1	373	17.6	759	17.9	113	23.8	110	23.5	223	23.6
Impulsivity score^a^												
Mean score	2080	2.24	2080	2.24	4160	2.24	497	2.3	497	2.3	994	2.3
SD		0.87		0.87		0.87		0.92		0.92		0.92

a These variables were measured at wave 1 only and therefore, data on the sample characteristics are the same for both wave 1 and wave 2. They are treated as fixed effects in the subsequent models.

**Table 2:** Gambling behaviour.

	*a. All participants*						*b. Those who gambled in both waves*					
	*Wave 1*		*Wave 2*		*Combined*		*Wave 1*		*Wave 2*		*Combined*	
	*n*	*%*	*n*	*%*	*n*	*%*	*n*	*%*	*n*	*%*	*n*	*%*
Individuals who undertook each in the past 12 months												
Lotteries	356	17.6	338	15.6	694	16.6	253	49.3	266	50.3	519	49.8
Scratchcards	371	18.9	285	14.0	656	16.4	255	50.2	227	45.2	482	47.7
Private betting	183	10.4	96	5.6	279	8.0	104	22.9	63	15.1	167	19.0
Fruit/slot machines	120	6.4	60	3.2	180	4.8	79	17.1	44	10.0	123	13.5
FOBT	36	2.3	21	1.1	57	1.7	24	5.9	15	3.2	39	4.6
Online horse/dog betting	137	6.9	95	5.1	232	6.0	93	18.3	81	17.7	174	18.0
Online sports betting	207	11.8	166	9.2	373	10.5	150	34.2	138	30.8	288	32.5
Online casino/slots	72	3.9	74	4.1	146	4.0	54	11.9	57	12.8	111	12.4
Online bingo	36	1.8	40	2.0	76	1.9	28	5.4	30	6.2	58	5.8
Online other betting	83	4.5	59	3.4	142	4.0	64	13.9	48	11.7	112	12.8
Horse/dog racing (in person)	127	6.5	31	1.6	158	4.0	81	16.5	28	5.7	109	11.1
Sports betting (in person)	43	2.5	26	1.6	69	2.1	31	7.2	22	5.7	53	6.4
Other form of betting (in person)	18	1.0	10	0.6	28	0.8	13	2.6	10	2.2	23	2.4
Casino table games (in person)	54	2.8	19	1.2	73	2.0	38	7.8	16	3.6	54	5.7
Bingo (in person)	124	5.3	36	1.5	160	3.4	77	13.0	31	5.2	108	9.1
Poker	20	1.1	25	1.4	45	1.3	13	3.0	23	5.4	36	4.2
Football pools	42	2.8	35	2.1	77	2.5	27	6.7	22	5.4	49	6.0
Loot boxes	214	12.1	196	10.7	410	11.4	84	18.1	76	17.1	160	17.6
Skin betting (any)	112	6.9	83	4.7	195	5.8	52	11.7	38	9.7	90	10.7
Skin betting (external websites)	73	4.2	59	3.1	132	3.6	35	7.3	29	6.8	64	7.0
Skin betting (private)	94	5.8	75	4.4	169	5.1	48	10.9	37	9.6	85	10.2
Number of activities in past 12 month												
None	1240	56.6	1391	66.0	2631	61.3	0	0	0	0	0	0
1	343	17.8	325	15.3	668	16.5	162	32.3	213	41.5	375	36.9
2	233	12.2	161	8.1	394	10.1	136	28.2	113	22.5	249	25.3
3	96	4.8	95	4.6	191	4.7	62	11.3	78	14.6	140	12.9
4	74	3.5	48	2.7	122	3.1	60	11.1	41	9.1	101	10.1
Five or more	94	5.3	60	3.4	154	4.3	77	17.1	52	12.3	129	14.7
Gambled on at least one activity in past 12 months	840	43.4	689	34	1529	38.7	497	100	497	100	994	100
PGSI score												
Mean score	2080	0.7	2080	0.6	4160	0.6	497	2.1	497	1.8	994	1.9
SD		2.84		2.57		2.71		4.79		4.34		4.57

FOBT = fixed odd betting terminals; PGSI = Problem Gambling Severity Index

**Table 3:** Zero-inflated negative binomial models: relationship between past year participation in gambling activities at wave 1 and PGSI scores at wave 2.

*Count model*	*Incidence-rate ratio*	*SE*	*95% CI (lower)*	*95% CI (upper)*	*P value*
PGSI score wave 1	1.17	0.02	1.13	1.22	<0.001
Lotteries	0.85	0.21	0.53	1.37	0.510
Scratchcards	1.04	0.21	0.70	1.55	0.834
Private betting	0.56	0.12	0.36	0.86	0.008**
Fruit/slot machines	0.92	0.22	0.58	1.48	0.736
FOBT	0.63	0.28	0.26	1.48	0.287
Online horse/dog betting	1.14	0.38	0.59	2.21	0.699
Online sports betting	1.34	0.35	0.81	2.22	0.257
Online casino/slots	1.20	0.36	0.66	2.16	0.550
Online bingo	1.20	0.29	0.74	1.92	0.460
Online other betting	1.22	0.40	0.64	2.32	0.548
Horse/dog racing	0.82	0.18	0.53	1.27	0.379
Sports betting	1.61	0.58	0.80	3.25	0.182
Other form of betting	2.51	1.22	0.97	6.49	0.057
Casino table games (in person)	1.10	0.28	0.67	1.80	0.720
Bingo (in person)	0.94	0.24	0.57	1.55	0.809
Poker	0.66	0.28	0.29	1.51	0.328
Loot boxes	1.08	0.30	0.62	1.87	0.796
Skin betting (any)	1.01	0.30	0.56	1.82	0.977
Control variables:					
Number of activities in past 12 months (baseline = 0 or 1)					
2 or 3	0.39	0.14	0.19	0.79	0.009**
4 or 5 more	0.34	0.15	0.14	0.81	0.015*
5 or more	0.26	0.13	0.10	0.67	0.005**
Constant	2.90	0.64	1.89	4.46	0.000**
***Inflate model***	***Coefficient***	***SE***	***95% CI (lower)***	***95% CI (upper)***	***P value***
Impulsivity scale	-0.24	0.14	-0.51	0.02	0.071
Male	-0.78	0.24	-1.25	-0.31	0.001**
Age in years	-0.01	0.06	-0.13	0.11	0.892
Non-White ethnic background	-0.15	0.34	-0.83	0.52	0.658
Personal income (baseline = <£5 k)					
£5 k-£19 999	-0.18	0.32	-0.81	0.44	0.566
£20 k+	-0.17	0.40	-0.96	0.61	0.666
Missing	0.01	0.26	-0.51	0.52	0.985
Individual has a degree	0.50	0.34	-0.16	1.16	0.138
Number of activities in past 12 months (baseline = 0 or 1)					
2 or 3	-1.88	0.35	-2.58	-1.19	0.000**
4 or 5 more	-3.58	1.70	-6.90	-0.25	0.035*
5 or more	-17.50	1.39	-20.23	-14.77	0.000**
Constant	3.26	1.19	0.94	5.58	0.006**
Log α	0.84	0.25	0.36	1.33	0.001**
α	2.32	0.58	1.43	3.78	

* P < 0.05.

** P < 0.01.

FOBT = fixed odd betting terminals; PGSI = Problem Gambling Severity Index; SE = Standard Error

**Table 4A:** Negative binomial regression of PGSI scores (full model).

	*Incidence-rate ratio*	*SE*	*95% CI (lower)*	*95% CI (upper)*	*P value*
Wave	1.13	0.220	0.78	1.66	0.514
Lotteries	1.07	0.182	0.76	1.49	0.705
Lotteries*wave interaction	0.51	0.106	0.34	0.76	0.001**
Scratchcards	1.08	0.175	0.78	1.48	0.654
Scratchcards*wave interaction	0.98	0.191	0.67	1.44	0.934
Private betting	0.81	0.167	0.54	1.22	0.314
Private betting*wave interaction	0.94	0.254	0.55	1.59	0.805
Fruit/slot machines	1.54	0.299	1.05	2.25	0.027*
Fruit/slot machines*wave interaction	0.91	0.238	0.55	1.52	0.726
FOBT	2.15	0.600	1.24	3.71	0.006**
FOBT*wave interaction	1.51	0.536	0.75	3.03	0.249
Online horse/dog betting	2.03	0.404	1.37	3.00	<0.001**
Online horse/dog betting*wave interaction	0.71	0.171	0.44	1.14	0.154
Online sports betting	0.91	0.160	0.64	1.29	0.593
Online sports betting*wave interaction	1.38	0.313	0.89	2.16	0.150
Online casino/slots	1.51	0.301	1.03	2.23	0.037*
Online casino/slots*wave interaction	1.27	0.310	0.79	2.05	0.332
Online bingo	0.88	0.231	0.53	1.47	0.637
Online bingo*wave interaction	1.54	0.481	0.83	2.84	0.169
Online other betting	1.39	0.256	0.97	2.00	0.071
Online other betting*wave interaction	1.36	0.363	0.80	2.29	0.256
Horse/dog racing	1.11	0.208	0.77	1.60	0.575
Horse/dog racing*wave interaction	1.11	0.377	0.57	2.16	0.764
Sports betting	0.72	0.175	0.44	1.16	0.175
Sports betting*wave interaction	1.15	0.413	0.57	2.33	0.689
Other form of betting	1.13	0.329	0.64	2.00	0.672
Other form of betting*wave interaction	1.39	0.715	0.51	3.81	0.526
Casino table games (in person)	0.94	0.246	0.57	1.57	0.827
Casino table games (in person)*wave interaction	1.11	0.434	0.52	2.39	0.782
Bingo (in person)	0.87	0.178	0.58	1.30	0.487
Bingo (in person)*wave interaction	0.75	0.297	0.34	1.63	0.463
Poker	1.99	0.552	1.15	3.43	0.013*
Poker*wave interaction	0.48	0.173	0.24	0.97	0.042*
Football pools	0.94	0.261	0.54	1.62	0.820
Football pools*wave interaction	2.02	0.825	0.91	4.50	0.084
Loot boxes	1.58	0.312	1.07	2.33	0.020*
Loot boxes*wave interaction	0.86	0.237	0.50	1.47	0.577
Skin betting (any)	2.14	0.468	1.40	3.29	<0.001**
Skin betting (any)*wave interaction	0.61	0.205	0.32	1.18	0.140
Control variables:					
Number of activities in past 12 months (baseline = 1 or 2)					
3 or 4	1.56	0.279	1.10	2.22	0.012*
5 or more	0.97	0.329	0.50	1.89	0.932
Impulsivity scale	1.54	0.124	1.31	1.80	<0.001**
Male	1.12	0.174	0.83	1.52	0.453
Age in years	0.94	0.035	0.87	1.01	0.100
Non-White ethnic background	2.11	0.489	1.34	3.32	0.001**
Individual is not in education, employment or training	1.05	0.183	0.75	1.48	0.772
Personal income (baseline = <£5 k)					
£5 k-£19 999	1.34	0.242	0.94	1.91	0.107
£20 k+	1.50	0.301	1.01	2.22	0.045*
Missing	1.20	0.226	0.83	1.73	0.335
Individual has a degree	0.74	0.113	0.55	1.00	0.048*
Constant	0.91	0.736	0.19	4.44	0.907

* P < 0.05.

** P < 0.01.

FOBT = fixed odd betting terminals; SE = Standard Error

**Table 4B:** Model statistics for Table 4a.

	*Parameter*	*SE*	*95% CI (lower)*	*95% CI (upper)*
ln_r	0.85	0.129	0.59	1.10
ln_s	0.06	0.186	-0.31	0.42
Likelihood test vs pooled sample: χ^2^ (0,1)	96.50			
Prob ≥ χ^2^	<0.001			

Abbreviation: SE, Standard Error.

**Table 5A:** Negative binomial regression of PGSI scores (parsimonious model).

	*Incidence-rate ratio*	*SE*	*95% CI (lower)*	*95% CI (upper)*	*P value*
Wave	1.02	0.1	0.79	1.32	0.870
Lotteries	0.84	0.1	0.63	1.12	0.237
Lotteries*wave interaction	0.69	0.1	0.49	0.97	0.032*
Fruit/slot machines	1.43	0.2	1.07	1.91	0.015*
FOBT	2.21	0.4	1.61	3.05	<0.001**
Online horse/dog betting	1.53	0.2	1.17	2.00	0.002**
Online casino/slots	1.26	0.2	0.92	1.73	0.145
Online casino/slots*wave interaction	1.61	0.3	1.12	2.30	0.009**
Online other betting	1.44	0.2	1.11	1.89	0.007**
Skin betting (any)	2.32	0.4	1.69	3.19	<0.001**
Control variables:					
Number of activities in past 12 months (baseline = 1 or 2)					
3 or 4	1.75	0.2	1.36	2.25	<0.001**
5 or more	1.41	0.3	0.96	2.09	0.083
Impulsivity scale (taken from W1)	1.54	0.1	1.33	1.79	<0.001
Male	1.09	0.2	0.82	1.45	0.554
Age in years	0.94	0.0	0.88	1.01	0.097
Non-White ethnic background	2.09	0.5	1.35	3.22	<0.001**
Individual is not in education, employment or training	1.03	0.2	0.76	1.40	0.846
Personal income (baseline = <£5 k)					
£5 k-£19 999	1.24	0.2	0.90	1.72	0.191
£20 k+	1.36	0.3	0.95	1.97	0.095
Missing	1.24	0.2	0.88	1.76	0.223
Individual has a degree	0.79	0.1	0.61	1.02	0.075
Constant	0.86	0.7	0.19	3.96	0.844

* P < 0.05.

** P < 0.01.

FOBT = fixed odd betting terminals; PGSI = Problem Gambling Severity Index; SE = Standard Error

**Table 5B:** Model statistics for Table 5a.

	*Parameter*	*SE*	*95% CI (lower)*	*95% CI (upper)*
ln_r	0.75	0.1	0.52	0.99
ln_s	0.12	0.2	-0.23	0.47
Likelihood test vs pooled sample: χ^2^ (0,1)	106.48			
Prob ≥ χ^2^	<0.001			

Abbreviation: SE, Standard Error.

## Footnotes

* Exceptions to this were National Lottery and Scratchcards, which at the time of data collection were legally available to those ages 16 and over.
