# Supplementary material for: The relationship between problematic gambling severity and engagement with gambling products: Longitudinal analysis of the Emerging Adults Gambling Survey
Source: Addiction. Author manuscript; Available in PMC 2023 Jul 13. (PMC7614752; doi:10.1111/add.16125)
Supplement: S1 [file EMS177458-supplement-S1.docx]

Appendix A: Treatment of missing values in analysis

This document sets out the number of missing values for each variable included in the analysis and the treatment applied to those values within the modelling. Analysis is based on those who took part in both waves (n=2080), thus missing values are presented for this group.

| **Variable** | **Number of missing cases** | **Treatment of missing cases** |
| --- | --- | --- |
| Age | 0 | N/A |
| Sex | 0 | N/A |
| Ethnic group | 93 | Recoded to modal value “White” |
| Tenure | 0 | N/A |
| Marital status | 80 | Recoded to modal value “single” |
| Employment status | 0 | N/A |
| Educational attainment | 142 |  |
| Parental education | 124 | Coded to dummy category “educational status unknown” |
| Personal income | 723 | Coded to dummy category “income unknown” |
| Region | 15 | Recoded to modal value “London” |
| Area Deprivation | 169 | Coded to dummy category “area deprivation unknown” |
| Impulsivity | 0 | N/A |
| Problem Gambling Severity Index Score | 0 | N/A |
| Lottery | 0 | N/A |
| Scratchcards | 0 | N/A |
| Private Betting | 0 | N/A |
| Slot machines | 0 | N/A |
| Fixed odd betting terminals | 0 | N/A |
| Online horse/dog betting | 0 | N/A |
| Online sports betting | 0 | N/A |
| Online betting other | 0 | N/A |
| Online casino/slots | 0 | N/A |
| Online bingo | 0 | N/A |
| Horse/dog racing at a bookmakers | 0 | N/A |
| Sport betting at a bookmakers | 0 | N/A |
| Other betting at a bookmakers | 0 | N/A |
| Casino table games | 0 | N/A |
| Bingo at a club/hall | 0 | N/A |
| Poker | 0 | N/A |
| Loot boxes | 30 | Recoded to modal value “Never” |
| Skin Betting | 47 | Recoded to modal value “Never” |
