# Supplementary material for: The relationship between problematic gambling severity and engagement with gambling products: Longitudinal analysis of the Emerging Adults Gambling Survey
Source: Addiction. Author manuscript; Available in PMC 2023 Jul 13. (PMC7614752; doi:10.1111/add.16125)
Supplement: S2 [file EMS177458-supplement-S2.docx]

**Appendix B: generation of longitudinal weights between wave 1 and wave 2 of the Emerging Adults Gambling Survey**

**Methodology**

To generate the longitudinal weights we build a model that predicts which people are likely to drop out between wave 1 and wave 2. We then upweight people who are *likely* to drop out (based on the predicted value), but do not actually dropout. Below are the steps followed to generate the attrition weights used in this study:

1. Take the Wave 1 data, and add an attrition variable that tells us whether this respondent answered wave 2 (coded 0 = answered, 1 = did not answer)
2. Run a logistic regression with “attrition variable” as the dependent variable, and the “candidate variables” as predictor variables (see below for details). We then generate predicted probabilities from the regression modelling
3. Drop cases from the datafile who did not respond at Wave 2
4. Divide each predicted value by the mean predicted value after dropping Wave 2 cases (for example, if respondent has a predicted probability of 0.375, and the mean predicted probability is 0.25, then 0.375 / 0.25  = 1.5. This is the **attrition weight**
5. Multiply the Wave 1 weight (which matched the responding sample in Wave 1 to the age, sex, deprivation and regional profile of young people living in Great Britain) by the attrition weight. This will be the Wave 2 **longitudinal weight.** This is the weighting variable used in this analysis.

**Summary**

The logistic modelling conducted in this study is based on these attrition predictor variables collected at Wave 1:

**Variable Name Description**

W1sex Gender, coded Male/Female

W1AgeBands Age (grouped), coded 16-18, 19-21, 22-24

W1ethnicg Self-reported ethnic identity (grouped), coded White/White British; Mixed; Asian/Asian British; Black/Black British; Other

W1neet Whether in Employment, Education or Training, coded yes/no

W1anystudent Whether currently in higher education, coded, yes/no

W1qimd Area Deprivation Decile, coded from least to most deprived

W1gamfreq2 Frequency of gambling on any activity, coded more than weekly; about weekly; about fortnightly; about monthly; a couple of times a year; did not gamble

W1anyacty Whether gambled on any activity in the past year or no, coded yes/no

W1ngamyr Number of gambling activities undertaken in the past year, range from 0 to 15

W1pgsiprob Problem Gambling Severity Index status, coded 0 = non- gambler/non-problem gambler; 1-2 low risk gambling; 3-7 moderate risk gambling; 8+ problem gambling.

Four variables were significant in the model result: W1sex (Gender) , W1ethnicg (Ethnicity), W1qimd and W1gamfreq2. The model shows that females were less likely to drop out than their male counterparts. Participants of Black/Black British origin were less likely to drop out than those from White/White British backgrounds. Those whose area deprivation status was unknown were more likely to drop out than those where this was categorized. Those who gambled at different levels of frequency were more likely to drop out than those who did not gamble. This was particularly evident for those gambling fortnightly.
